# Bibliographical Notices

**Published:** 1855-04

**Authors:** 


### EDITORIAL DEPARTMENT.

### BIBLIOGRAPHICAL NOTICES.

*What to observe at the Bed Side and after Death in Medical Cases. Published under the authority of the London Medical Society of Observation. Second American, from the second and enlarged London edition.* Philadelphia. Blanchard & Lea, 1855.

Two years only have elapsed since the publication of the first edition of this little manual of clinical observation, and already the demand has exhausted it. This proves its applicability to the purposes for which it was intended, and the general sense of the necessity of such a guide.

It originated with the London Medical Society of Observation, an association established for the purpose of studying and comparing clinical facts. It was found that the labor of classification would be materially diminished, and the comparison of observations greatly facilitated, if all clinical reports were made out upon one definite plan. Dr. Walshe having drawn up a form of arrangement of symptoms, it was adopted by the association, and, after being modified by a committee of publication, assumed its present shape.

The examination recommended is very minute, and of so searching a nature as to render it almost impossible for any appreciable symptom to escape the observer. At the same time, it is so extended as to require no ordinary patience on the part of the physician and no little docility on that of the patient to carry it out. Thus, under the head of "Personal Description and Peculiarities of the Patient in Health," we find: "*Sex:* degree in which sexual characters are marked (general conformation, *external sexual organs,* mammæ, voice, beard, &c.) sexual peculiarities; *natural force of instinct,*" &c. How this may do for a hospital in Paris or London, we are not prepared to say, but we pity the unfortunate doctor who, with this manual in his hand, should endeavor to subject all his lady patients to its strict requirements. Setting aside this fault of excessive fullness and over-anxiety to exhaust the subject, the book is an excellent one. It directs the attention of the inexperienced practitioner to symptoms that might otherwise have escaped him, and aids even the fullest experience by acting as a memorandum of observations.

---

*Transactions of the American Medical Association,* Vol. Vii. New York. Charles B. Norton, 1854.

THESE transactions retain the interest which attached to their predecessors. The present volume contains numerous full epidemiological reports, and much valuable information in regard to medical topography, geology and meteorology.

The report of Dr. F. Peyre Porcher, of Charleston, S. C., is, as might be expected, full, learned and satisfactory. It is an exhaustive study of the properties, medical, toxicological and economical, of the numerous ***cryptogamic plants*** of our country.

Dr. Fenner's ***report on the Epidemics of Louisiana, Mississippi, Arkansas and Texas*** for 1853, possesses a terrible interest to the reader because that was the year in which yellow fever so frightfully scourged the region under consideration.

The prize essay of Dr. Brainard, on a ***New Method of Treating Disunited Fractures aud certain Deformities of the Osseous System,*** contains a number of physiological facts concerning the bones, and the account of numerous experiments illustrating the action of various substances upon them. Several cases, illustrating the value of the treatment of these fractures by perforating the bones, are given, but the readers of the medical journals have already seen the majority of them in print. The report is accompanied by handsomely executed lithographic plates.

The report on *Medical Education* from the pen of Dr. Cabell, is an echo of the profound dissatisfaction of thinking men with the present degraded condition of the standard of medical requirements. Even the feeble requisitions of the first convention in 1846, that the candidate for medical honors should have a preliminary education equal to that of the shop-boy in a druggist's establishment, have been neglected, and the profession has the mortification of seeing its standard lowered, year after year, by a perpetual influx of stupidity.

The schools are the same humbugs that they always were. Huckstering for students, peddling in diplomas, puffing in the papers, organizing sham clinics, bragging about imaginary hospital advantages, they secure their fees, and disembogue their trash upon the profession. The existing medical education is, as every one must confess, altogether insufficient, and the present report says so. Unfortunately, however, the report was not read, nor were the resolutions appended to it adopted by the association, on account of the absence of its author.

We were not a little amused at the prominence given to the University of Virginia, in this report, and yet justice compels us to say that the young men who come from that school to the cities, are generally remarkable for their attention to their studies, their intelligence, and their zeal in availing themselves of the practical advantages there furnished.

The paper recommends a plan we have often advocated in this Journal, the divorce of the teaching and licensing powers. The corruption arising from their union is too well known to need any comment.

But we can no longer dwell upon this subject, for the theme expands before us. We design to make some further remarks upon medical education at a future time, and shall reserve what we have to say, for that occasion.

---

*Proceedings and Reports of the Medical and Chirurgical Faculty of Maryland for the year* 1854. Published by order of the Faculty. Baltimore, 1855.

We find in this pamphlet two interesting reports, one on Surgery, by Dr. C. Johnston, the other on Medical Chemistry, by Dr. C. Frick.

The first commences with a vindication of the microscope and an account of its application to the classification of pathological growths. It then proceeds to consider the modifications which the progress of observation has effected in our views of the process of repair in broken bones. Aneurism is next taken up and we have a valuable table of the results of the various modes of treatment, ligature, compression, electro-puncture and injection with perchlorid of iron. The report closes with an account of the various modifications of Helmholtz's eye speculum. This report is accompanied with a neat wood cut printed in colors, illustrating the appearance of the eye in health, and in incipient cataract, as seen through the instrument in question.

The report on Medical Chemistry discusses briefly the pathology of diabetes, as illustrated by Bernard's discovery of the sugar-making function of the liver and the observations of Robin and Verdeil, Henle and others upon its relation to the respiration, &c. Recent advances in the chemistry of the urine are then noticed, and the attempts of Bence Jones to dissolve urinary calculi in the body are dwelt on at some length.

The processes of Stas and Flandin for the vegetable alkaloids are then briefly alluded to, the preference being given to that of Flandin. We have employed this method ourselves in medico-legal researches for organic poisons, but were not satisfied with it. It is far better than the old sulphuric acid process, but it still leaves much to be desired.

The tests of strychnia of Day and Lefort are both given. Lefort's test is very decidedly interfered with by the presence of organic matters, so that when even a small quantity of fat is present, no violet tint can be perceived but only the dirty green of hydrated oxyd of chromium. Our own preferences are decidedly for Day's test, viz. ferricyanide of potassium and sulphuric acid. When mixed with strychnia, they give a rich violet hue, rapidly changing to a brick red which is quite as characteristic as the former tint. The latter is produced even when a large quantity of fat is mixed with the alkaloid.

The report closes with an account of some experiments made by its author on patients in the Maryland Penitentiary. Without altering the treatment in any given case for the sake of the observations, he nevertheless has succeeded in obtaining some excellent results. Having noted the quantity and solid contents of the urine of persons taking no medicine, he proceeded to investigate the influence of various remedies upon this secretion. He found that a mixture of the sulphate of quinine and of iron increased the solid contents of the urine 60 per cent., while cod liver oil decidedly diminished them.

We regret that we have no space for a fuller abstract of these interesting reports, and we recommend them to our readers as well worth a careful perusal.

---

*Report of the Superintendent of the Coast Survey for* 1853. Washington, 1854.

We have received from the Coast Survey Office this valuable public document. Few who have not looked into these volumes have any idea of the amount of interesting and valuable information they contain.

Besides the main business of exploring the coast for nautical purposes, the Coast Survey is continually developing important scientific facts. In the present report we have accounts of the microscopic character of the ocean bottom in the Gulf stream, of the tides in various parts of our country—of analyses of boiler crusts—of computations of longitude and other astronomical observations, and descriptions of various pieces of apparatus and important processes in the arts.

Add to this a set of maps which exhibit not only the hydrography of our coast, but the progress of the survey for each successive year, and there is here a mine of valuable knowledge which cannot be easily given up, when its richness has once been ascertained.

